# CBAM-DenseNet with multi-feature quality filtering: advancing accuracy in small-sample iris recognition

**DOI:** 10.3389/frai.2025.1714882

**Published:** 2026-01-27

**Authors:** Yongheng Pang, Zishen Wang, Nan Jiang, Jia Qin, Suyuan Li

**Affiliations:** 1 Shanghai Key Laboratory of Forensic Medicine and Key Laboratory of Forensic Science, Ministry of Justice, Shenyang, Liaoning, China; 2 School of Public Security Information Technology and Intelligence, Criminal Investigation Police University of China, Shenyang, Liaoning, China

**Keywords:** attention mechanism, CBAM, deep learning, DenseNet, image quality assessment, iris recognition, multi-feature fusion

## Abstract

In the context of the information age, traditional password and key-based authentication mechanisms are no longer sufficient to meet the growing demands for information security. Iris recognition technology has garnered attention due to its high security and uniqueness. Current iris recognition methods based on single feature extraction are prone to loss of feature information, which affects recognition rates. To address this, this paper proposes a multi-feature fusion-based iris recognition method. The method employs a comprehensive quality evaluation scheme to filter iris images, ensuring the quality of the input images. An improved CAN network is used to effectively remove image noise, and a DenseNet network-based iris feature extraction method is combined with a fusion space and attention mechanism (CBAM) to enhance the expressiveness of features. Through experiments with small sample sizes and testing on various public iris databases, the proposed method has been validated for significant improvements in recognition accuracy and robustness.

## 1 Introduction

With the rapid advancement of informatization and networking, the demand for information security and personal identity verification in society is growing rapidly, posing an urgent need for high-standard identity authentication mechanisms. Traditional methods of password and key authentication are no longer sufficient to meet current security challenges. Biometric recognition technology, with its efficiency and reliability, has gradually become a hot topic in research and application in the field of identity authentication to meet the increasing security needs. The widespread adoption of biometric recognition technology in identity frameworks across various industries is becoming more prevalent (Gupta, 2023). Iris recognition technology (IR), with its high level of security and difficulty to forge, has become one of the most watched and researched technologies in recent years (He and Li, 2024). The iris is the annular pigmented membrane located between the pupil and the sclera of the eyeball, which appears to have a radially textured pattern from the inside out, along with interspersed spot textures (Kim et al., 2023). Through extensive analysis of the anatomical structure of the iris by ophthalmologists and anatomists, it has been found that the iris, similar to fingerprints, possesses a unique individuality that is distinct from others, and even identical twins have different irises (Wei et al., 2022). Based on the inherent structure of the iris, it offers more accurate and reliable advantages compared to other biometric features such as facial, fingerprint, voice, gait, and vein recognition (Zambrano et al., 2022). Therefore, iris recognition has become a hot topic in biometric recognition research in recent years and is one of the most promising identification technologies. The first step in iris recognition is to evaluate the quality of the collected iris images and select qualified images. Daugman (2001) judged the clarity of the image based on the high-frequency energy of the 2D Fourier of the iris. Wildes (1997) evaluated the roughness of the image by measuring the grayscale changes at the contour of the iris and sclera. However, a unidimensional iris image quality evaluation method obviously cannot meet the application standards of iris quality evaluation in practical applications. Therefore, (Gao et al., 2015) adopted a coarse and fine combined indicator, based on Support Vector Machine (SVM) to achieve the fusion of image quality indicators, and used this to distinguish images of different qualities; (Wang et al., 2020) proposed a recognition-oriented quality assessment method, using the distance of the image embedded in the feature space as a quality indicator, and predicted based on a deep neural network with an attention mechanism. This method significantly improved the performance of the recognition algorithm. Iris localization is also key to iris recognition. In the early days, (Daugman, 2003) used a circular template matching method to achieve the localization of the iris in the human eye. With the development of computer vision theory, (Wildes, 1997) proposed using Hough transform to outline the iris contour boundary to achieve iris localization in the human eye, which has been used to this day. Subsequently, with the continuous development of machine learning and deep learning theories, (Ishikawa, 2004) proposed an active appearance model-based eye localization algorithm, which determines the position of the iris in the human eye by describing the entire external structure of the face. Jan et al. (2021) proposed an iris localization algorithm based on four steps: preprocessing, marking rough pupil area, extracting rough eye contour, and refining eye contour, which effectively localized the iris area and removed noise. With the development of deep learning, significant breakthroughs have been made in both application and theoretical aspects of iris recognition technology over the past few decades. Currently, mainstream feature extraction algorithms for iris feature extraction and matching are based on the description of iris texture. Boles utilized an algorithm based on wavelet transform and zero-crossing point detection for feature extraction of irises to be matched, and encoded by the average of the integral between adjacent zero-crossing points to obtain the corresponding iris feature template (Boles and Boashash, 1998). Wildes et al. (1994) to obtain iris texture images at different scales as features, constructing iris feature templates. However, traditional single-feature iris features have the problem of insufficient feature extraction and low accuracy. With the development of deep learning, deep learning-based iris feature extraction can extract iris features very well. Gangwar and Joshi (2016) proposed a DeepIrisNet neural network, which has a deeper network structure and can be well applied to large-scale iris datasets, and based on the simulation of iris microstructure, it achieves high recognition accuracy. Al-Waisy et al. (2018) proposed a deep learning neural network to distinguish between left and right irises, and the features learned by this neural network model are fused based on the hierarchical fusion method, which improves the accuracy and speed of iris recognition. A bit-parallel event matching framework leveraging AVX-512 vectorization has demonstrated over 35 × speedup by encoding event streams into time-sliced bit sequences and exploiting SIMD parallelism for complex temporal pattern evaluation (Qiu et al., 2025). However, in the specific practical application of iris recognition systems, existing iris recognition systems still face many adjustments in key links such as iris image quality evaluation, iris image preprocessing, and iris image feature extraction (Tan et al., 2012; Kaur et al., 2010; Ahmadi and Akbarizadeh, 2018; Zhou et al., 2020). The existing research difficulties mainly include how to ensure the improvement of iris recognition model performance, how to build efficient and universal recognition models based on different iris databases, and how to adjust and transform the internal structure of neural network recognition models to adapt to the specific needs of iris recognition, and other series of problems. Therefore, in response to the above problems, this paper proposes a multi-dimensional feature fusion-based iris recognition method framework, and the main contributions are as follows:

1. A method for iris image quality evaluation and screening is proposed. This includes five processes: liveness detection of iris images, clarity evaluation, squint detection, annular area detection, and clarity evaluation of the annular area, and the evaluation results of each part are input into the SA-SVM classifier for quality screening.
2. A method for preprocessing of iris images is proposed. Through four steps of SE-CAN network noise reduction, iris image localization, normalization and ROI area selection, and iris image enhancement, the iris image is preprocessed to ensure accurate extraction of iris features.
3. A multi-dimensional feature fusion-based iris recognition method framework is proposed, covering the entire process of image evaluation screening, image preprocessing, feature extraction and multi-dimensional feature fusion, and identity recognition. It has been verified on public datasets such as CASIA v4.0, NICE1.0, and JLU-6.0, confirming the effectiveness, robustness, and generalization ability of the method.

The subsequent research work of this paper is arranged as follows: In the second section, the related indicators of iris image quality evaluation and preprocessing methods are proposed, and the evaluation results are input into the SA-SVM classifier for screening. In the third section, the strategy plan for iris image preprocessing is given. In the fourth section, the iris feature extraction method based on CBAM-DenseNet is introduced, and the multi-feature fusion strategy is designed. In the fifth section, the classification effect of the SA-SVM classifier and the fusion effect of the multi-feature fusion scheme are analyzed on multiple datasets. The proposed CBAM-DenseNet is compared with multiple networks in the experimental comparison, and the recognition effect of the network is evaluated. In the sixth part, a summary of the iris recognition process proposed in this paper is given.

## 2 Iris image quality evaluation and screening

### 2.1 Iris liveness detection

The dynamic characteristics of the natural constriction and dilation of the pupil provide a theoretical basis for liveness detection. This study employs linear arithmetic subtraction of iris images to verify the presence of dynamic expression within a sequence of iris images. By selecting the same visual organ from a specific individual and comparing differences in pupil coordinates and radius parameters under different intensities of illumination, the dynamic changes between adjacent images are assessed to achieve detection of iris vitality.

### 2.2 Iris image clarity assessment

The use of the Benner function calculates the overall clarity value of an iris image. The specific formula is as follows:


$$\begin{array}{l} F=\underset{x}{\sum }\underset{y}{\sum }{\left\{f\left(x+2,y\right)-f\left(x,y\right)\right\}}^{2} \end{array}$$ (1)

Accumulate the gradient values of all pixel points in the image to obtain the evaluation value *F* Where *x*, *y* are the grayscale values of adjacent pixels in two directions of the image. This evaluation value is used to screen iris images that meet quality standards. When *F* exceeds the preset threshold, the image is determined to be clearly captured; otherwise, it is determined to be a defocused and blurred iris image.

### 2.3 Iris image gaze detection

Calculate the ratio of the distance between the center of the iris pupil and the center of the iris image to the overall diagonal length of the iris image to reflect the degree of gaze deviation of the iris image. The detailed calculation method is as follows:


$$\begin{array}{l} L=\frac{\sqrt{{\left(x_{0}-x_{1}\right)}^{2}+{\left(y_{0}-y_{1}\right)}^{2}}}{\sqrt{m^{2}+n^{2}}} \end{array}$$ (2)

The ratio *L* reflects the degree of gaze deviation in the iris image, (*x*_0_, *y*_0_) represents the center position of the pupil in the iris image, (*x*_1_, *y*_1_) represents the center point position of the iris image, and *m* and *n* denote the length and width of the evaluated iris image, respectively. The greater the distance between the pupil center and the center of the iris image, the larger the corresponding ratio *L*, indicating a higher degree of iris deviation.

### 2.4 Iris image circular region detection

Iris image annular area detection is divided into two key parts: the detection of the area ratio of the iris annular region and the detection of the closure obstruction degree of the iris image. The area ratio of the iris annular region is represented by the ratio of the area of the pupil region in the iris image to the area ratio of the iris outer edge contour, reflecting the area ratio of the iris annular region. The relevant calculations are as follows:


$$\begin{array}{l} t_{1}=1-\Delta t=1-\frac{\partial_{1}}{\partial_{2}} \end{array}$$ (3)

Where ∂_1_ represents the area of the outer circular contour of the iris, ∂_2_ represents the size of the pupil area in the iris image, and *t*_1_ represents the quality evaluation coefficient. The detection of closure obstruction in iris images is primarily based on the assessment of the integrity of the pupil in the iris image. The formula is as follows:


$$\begin{array}{l} t_{2}=\frac{\beta_{1}}{\beta_{2}} \end{array}$$ (4)

Where β_1_ represents the total number of pixel points in the pupil area of the iris image, β_2_ represents the number of pixels with a grayscale value of 0 in the pupil area of the iris image, and *t*_2_ represents the degree of occlusion of the human eye open and close in the iris image. By performing the detection of the iris annular area ratio and the degree of eye closure occlusion on the iris image in sequence, the corresponding iris image quality evaluation coefficients *t*_1_ and *t*_2_ are obtained. These two evaluation coefficients are given different weight parameters *u*_1_ and *u*_2_ to be added and merged as the comprehensive quality evaluation index coefficient *T*_*r*_ of the iris annular area, see formula:


$$\begin{array}{l} T_{r}=u_{1}\times t_{1}+u_{2}\times t_{2} \end{array}$$ (5)

### 2.5 Iris image annular region clarity evaluation

Firstly, the clarity of the annular region of the normalized iris image is evaluated using the Benner (Equation 1) function-based image clarity evaluation function, obtaining the clarity function value *f*_1_. Secondly, the normalized iris image is processed for ROI (Region of Interest) extraction, denoted as *f*_2_, and the clarity of the extracted iris image ROI is evaluated using the Benner function, yielding the clarity function value. Finally, these two evaluation coefficients are weighted differently by parameters ω_1_ and ω_2_ and summed to form the clarity evaluation index coefficient for the iris annular region, denoted as *F*_*h*_. The specific calculation is as follows:


$$\begin{array}{l} F_{h}=\omega_{1}\times f_{1}+\omega_{2}\times f_{2} \end{array}$$ (6)

### 2.6 Iris image quality assessment based on simulated annealing-optimized support vector machine

**Figure 1:**
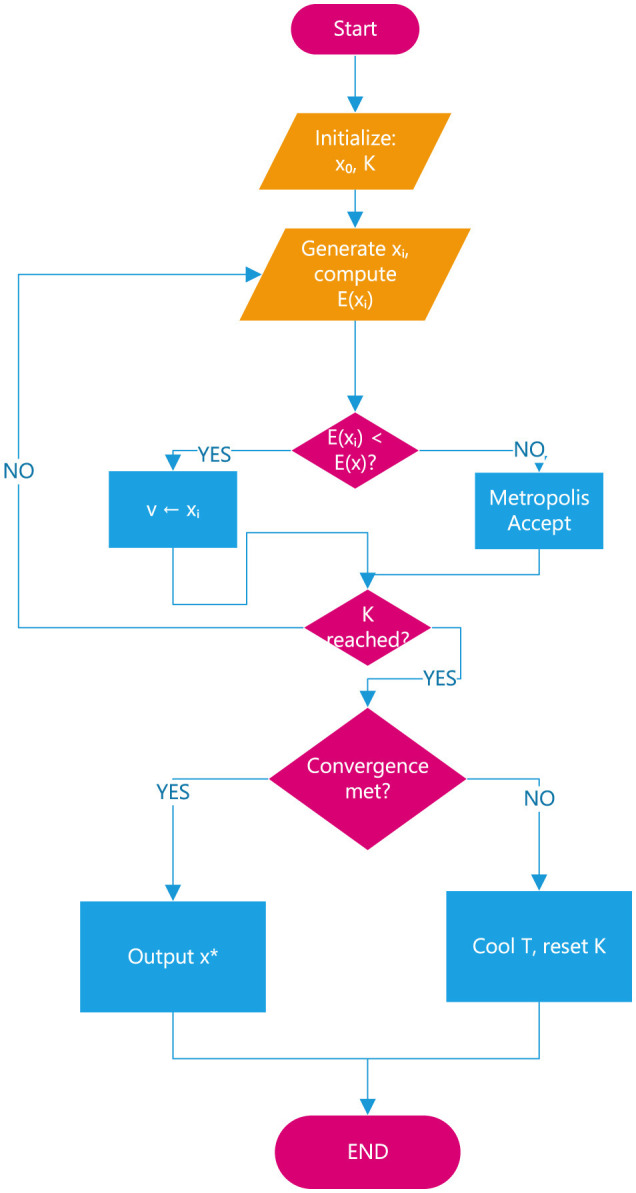
Flowchart of parameter optimization with the SA algorithm.

Integrate assessments of the iris image's off-angle, annular region, and ROI to obtain corresponding iris image quality evaluation metrics. Fusion of related iris quality rating coefficients establishes the iris quality evaluation vector {*L, T, F*_*h*_}. To reduce the impact of magnitude differences on classification accuracy and to enhance the speed of model training, standardize the image quality evaluation vector to obtain the standardized sample vector $\left\{L^{\prime },T^{\prime },F_{h}^{\prime }\right\}$. Input the obtained sample vector into the SA-SVM classifier for classification processing. Select appropriate kernel functions and optimal balance coefficients to improve classification accuracy. The Gaussian kernel is particularly suitable for iris image data with a small number of samples and low-dimensional sample vectors due to its mapping advantages in feature space. Furthermore, parameter optimization is performed using the Simulated Annealing (SA) algorithm to avoid falling into local optima and enhance the generalization capability of the SVM. This method can improve the performance of the SVM classifier and achieve better generalization under suitable penalty coefficients and kernel parameters. The specific process is depicted in Figure 1.

## 3 Iris image preprocessing

### 3.1 Iris image denoising based on the SE-CAN network

The CAN network is a fully convolutional neural network designed for learning and training variable resolution images, represented by multiple consecutive layers: {*I*_0_, *I*_1_, *I*_2_, …, *I*_*i*_, …, *I*_*j*_}. Here, *I*_0_ and *I*_*j*_ represent the input and output layers of the network, respectively, with the image dimensions at the input and output layers being the same. In the CAN network architecture, the spatial dimensions of each intermediate layer *I*_*i*_{0 < *i* < *j*} are *m* × *n* × *w*, where *m* × *n* is the resolution size of the image, and *w* is the number of mapped features. The image input to the network, after being processed through calculations, is passed to the next layer. The content transmitted to intermediate layer *I*_*i*_ is obtained by computing the output of layer *I*_*i*−1_. For specific computations, see the formula:


$$\begin{array}{lll} I_{i}^{a} & = & \Phi \left({\text{Ψ}}_{i}\left(g_{i}^{a}+\overset{j-1}{\underset{b=1}{\sum }}I_{i-1}^{b}{⊕}_{ra}K_{ij}^{a}\right)\right) \end{array}$$ (7)

$$\begin{array}{lll} {\text{Ψ}}_{i}\left(x\right) & = & \lambda_{i}x+\mu_{i}\text{BN}\left(X\right) \end{array}$$ (8)

**Figure 2:**
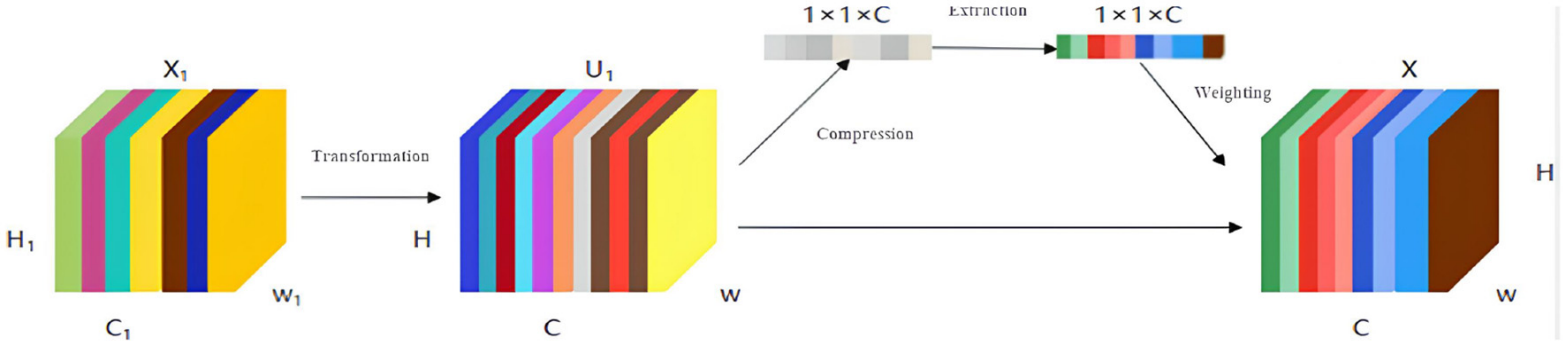
Workflow of the channel attention mechanism.

In the given formulas, $I_{i}^{a}$ and $I_{i-1}^{b}$ represent the feature maps *a* and *b* of the intermediate layer *I*_*i*_ and the previous layer *I*_*i*−1_, respectively. $g_{i}^{a}$ denotes the offset scalar, and $K_{ij}^{a}$ represents a 3*x*3 convolution kernel. The asterisk ⊕_*ra*_ signifies a dilated convolution. ψ_*i*_ indicates an adaptive normalization function, where λ_*i*_ and μ_*i*_ are learnable weights that are adjusted through backpropagation. ϕ denotes a nonlinear activation function operation. The processing procedure for bilateral filtering based on the CAN network includes: first, applying a bilateral filter to the training image samples and storing the results. Second, randomly extracting patches from the original sample images and those processed by the bilateral filter, and extracting data from the random patches to input into the network for training. Through the preset multi-scale CAN layers, the loss between the images processed by conventional filters and those processed by the CAN network is calculated. Finally, the CAN network is trained to approximate the bilateral filter operator based on the loss function. To enhance the correlation of feature information between different channels, a channel attention mechanism Squeeze-and-Excitation(SE) is introduced to the existing CAN network model, as shown in Figure 2.

**Figure 3:**
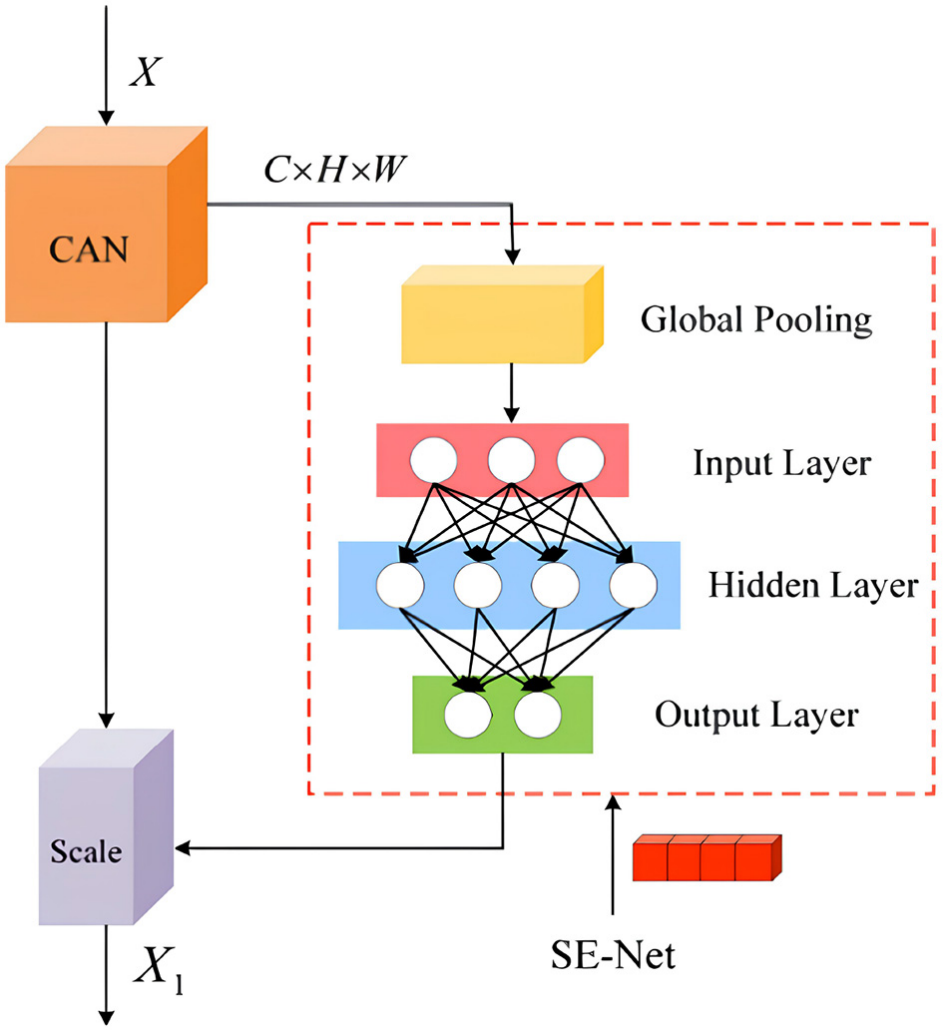
Structure of the improved SE-CAN network.

The integration of the channel attention mechanism forms the SE-CAN network, which ensures the preservation of important image features during processing and enhances the quality of reconstructed images after noise processing. Details of the SE-CAN network are shown in Figure 3.

The original image input to the convolutional layer is processed to generate feature map *X*1, which is transformed to obtain a new feature map *U*1 with a size of *H* × *W*. *U*1 contains *C* channels, and the importance of the images output by each channel varies, which may affect the learning and training effectiveness of the image. An attention mechanism module is introduced to perform global average pooling on each channel, and each channel obtains a scalar. Based on the channel attention mechanism, it is processed through two fully connected layers to obtain a scalar between 0 and 1 as the weight of that channel. Each element of the original image *H* × *W* is multiplied by the corresponding channel weight to obtain a new feature image *X*. Compared with the original network structure, the introduction of the channel attention mechanism module can effectively improve the network performance.

### 3.2 Iris image localization

To address the common noise types present in real-world iris images, such as Gaussian noise and salt-and-pepper noise, we propose an enhanced SE-CAN network. The improvement lies in the integration of a SE block within the CAN framework, which adaptively recalibrates channel-wise feature responses by explicitly modeling inter-channel dependencies. This attention mechanism allows the network to emphasize informative channels while suppressing those corrupted by noise, thereby enhancing the robustness of the extracted features. Specifically, the SE block performs a global averaging operation on the feature maps to generate a channel descriptor, followed by two fully connected layers that learn a nonlinear transformation to produce channel-wise weights. These weights are then applied to rescale the original feature maps, effectively refining the representation for subsequent processing stages. The iris image localization algorithm aims to identify the inner and outer edges of the iris, integrating morphological and Hough circle transform methods. The specific steps are as follows:

- Convert the iris image to a grayscale image and calculate its grayscale histogram. By analyzing the peaks and valleys of the histogram, determine the threshold for binarization to reduce interference from low grayscale values in the pupil area.
- Perform morphological circular closing operations on the iris image to remove interference such as eyelashes and eyelids, achieving noise reduction processing.
- Binarize the denoised iris image, use the Canny operator to perform edge detection on the image to extract the edge information of the pupil, and use the Hough circle transform to determine the radius size of the inner edge contour of the iris.
- Perform ROI cropping and binarization on the original image, set the outer edge threshold, and enhance the recognition of the outer edge contour through morphological circular closing operations.
- Integrate the above steps to accurately locate the iris, extract the inner and outer edge contour information, and provide an accurate iris area for subsequent image processing.

### 3.3 Iris image normalization and ROI area selection

Iris image normalization typically employs an elastic rubber circle model for iris normalization expansion. The steps are as follows:

- Utilize iris localization techniques to determine boundary information.
- Transform the iris boundary into a fixed-size rectangular area through polar coordinate mapping and radial transformation. Select areas that are rich in texture features and less affected by noise density.
- Determine the size and position of the ROI area based on the type of iris data.

### 3.4 Enhancement of iris images

Iris image enhancement typically utilizes histogram equalization technology, which involves converting the iris image to a grayscale image, computing its histogram, generating and normalizing the cumulative histogram, and applying a histogram equalization function to enhance contrast and details.

## 4 Iris feature extraction and fusion

### 4.1 Iris feature extraction based on CBAM-DenseNet

**Figure 4:**
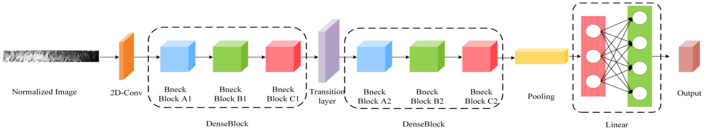
DenseNet network architecture.

Four different types of traditional single feature extraction algorithms (multi-channel Gabor filter algorithm, GLCM algorithm, Haar wavelet transform, and LBP algorithm) are used for feature extraction and classification. To effectively address the challenges of overfitting and poor generalization inherent in small-sample learning, our method incorporates three key strategies. First, the DenseNet architecture is inherently suitable for limited data due to its dense connectivity, which promotes feature reuse and mitigates the vanishing gradient problem, thereby enhancing model stability. Second, Dropout layers are strategically placed after each “BN-ReLU-Conv” block within the DenseBlocks and Transition-layers to serve as a regularization mechanism, randomly deactivating neurons during training to prevent co-adaptation. Third, systematic data augmentation is applied to the Region of Interest (ROI) images prior to training, including operations such as blurring, brightness adjustment, rotation, contrast variation, and flipping, to artificially expand the dataset and improve the model's robustness and generalization capability. Furthermore, a deep network model DenseNet and its convolutional network are employed for further feature extraction and fusion. DenseNet is composed of two types of core modules: DenseBlock and Transition-layer. The DenseBlock module facilitates inter-layer feature transfer through dense connections of multiple bottleneck modules. The Transition-layer module is used for dimensionality reduction and to decrease the size of the feature maps to prevent overfitting. Additionally, DenseNet incorporates convolutional and fully connected layers at the front and back ends, respectively, to expand the scope of feature extraction and optimize classification performance. For detailed structural information, please refer to Figure 4.

**Figure 5:**
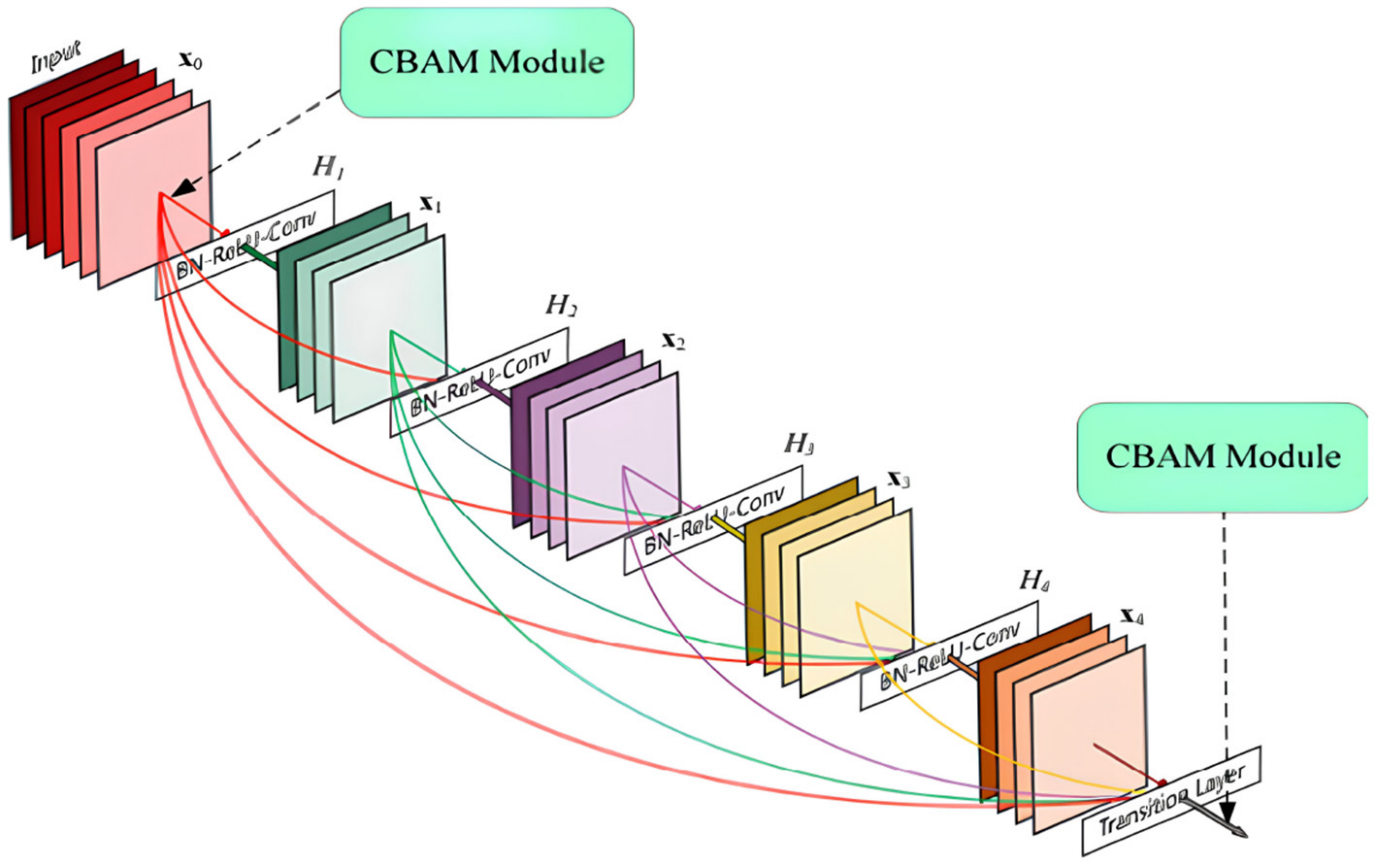
CBAM-densenet network architecture.

To enhance the model's ability to recognize key features, a Convolutional Block Attention Module (CBAM) fusion mechanism is introduced. The CBAM module optimizes feature retention and interference suppression by adaptively learning the channel and spatial features of the image. CBAM is integrated in front of each DenseBlock and after each Transition-layer to reduce computational complexity and the number of parameters. Figure 5 illustrates the DenseNet structure integrated with CBAM. To prevent overfitting, a Dropout layer is introduced after the “BN-ReLU-Conv” structure in DenseNet. Since each DenseBlock contains two such structures, two Dropout layers are added within each DenseBlock. However, as a dimension reduction layer, the Transition-layer only contains one “BN-ReLU-Conv” structure, hence only one Dropout layer is added.

**Figure 6:**
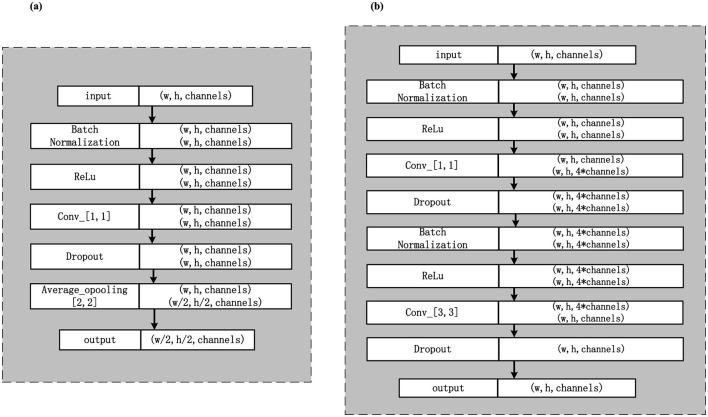
Optimization details of DenseNet network architecture. **(a)** Schematic diagram of DenseBlock structure optimization. **(b)** Schematic diagram of Transition-layer structure optimiztion.

Furthermore, to maintain the size of the feature map while introducing non-linearity and reducing the number of parameters, a 1x1 convolutional layer is added after the DenseBlock and Transition-layer. This not only enhances the network's discrimination and expression but also reduces computational load due to the small number of parameters, accelerating training. For details of the optimized network structure, please refer to Figure 6.

### 4.2 Assessment of multi-feature fusion strategies

The multi-feature fusion strategy for iris images aims to integrate various feature extraction techniques to generate a comprehensive feature vector, thereby optimizing iris recognition performance. When fusing, it is necessary to consider the characteristics of each feature method, as well as their intercorrelations and complementarities. Based on the characteristics of individual features, Gabor, CS-LBP, Haar wavelet features are combined with GLCM features to form four fusion schemes, as shown in Table 1. The proposed multi-feature fusion strategy integrates complementary information from four distinct texture descriptors. Specifically, Gabor filters are employed to capture frequency-domain textural patterns, CS-LBP (Completed Local Binary Pattern) and Haar wavelet transform are utilized to extract spatial-domain local micro-textures and multi-scale edge/contour features, respectively, while GLCM (Gray-Level Co-occurrence Matrix) is used to characterize global statistical textural properties such as homogeneity and contrast. To combine these heterogeneous features effectively, we adopt a channel concatenation mechanism. Each feature extraction method generates a single-channel feature map of size 32 × 256. These four maps are then stacked along the channel dimension to form a unified 4-channel fused feature tensor of size 32 × 256 × 4. This concatenated feature representation preserves the original characteristics of each individual descriptor without loss of information and serves as the input to the subsequent CBAM-DenseNet network for deep feature learning and classification. After feature extraction from iris images, a channel connection method is used according to the established fusion strategy to synthesize new feature images, which are then input into the CBAM-DenseNet model for training. Concurrently, the training process is statistically summarized to select the optimal fusion strategy based on relevant conditions during the learning process. Through the experimental analysis in Section 5.4, Scheme 4 demonstrated faster convergence, higher accuracy, and more stable performance during the training process, thus being chosen as the final feature fusion strategy.

**Table 1:** Comparison of multi-feature fusion schemes.

**Multi-feature fusion schemes**	**Fusion features**
Scheme 1	Gabor + Haar Wavelet + GLCM
Scheme 2	Haar Wavelet + CS-LBP + GLCM
Scheme 3	Gabor + CS-LBP + GLCM
Scheme 4	Gabor + Haar Wavelet + CS-LBP + GLCM

### 4.3 Recognition process based on multi-feature fusion

**Figure 7:**
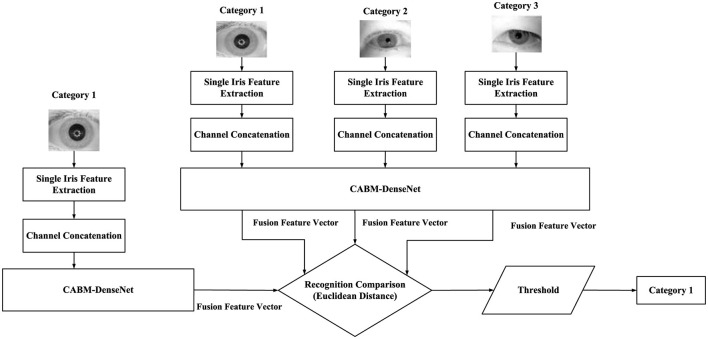
Multi-feature fusion iris recognition process.

The fused iris features are used for recognition, requiring the extracted features to be employed for iris recognition matching. By comparing the extracted feature vectors with known individual feature vectors to determine whether the similarity or distance between them reaches a certain threshold, authentication or verification can be carried out. Gabor frequency domain and CS-LBP spatial domain feature images, Haar wavelet mapped images, and GLCM single features from each iris are used. After channel splicing, they are adjusted to a size of 32 × 256 to form a comprehensive iris feature image, which is input into the CBAM-DenseNet network model for further feature extraction and fusion. The extracted feature vector is a multidimensional vector output by the fully connected layer. By calculating the Euclidean distance between the feature vectors of the known category label iris images and the feature vectors of the iris images to be recognized, and comparing it with a preset threshold, the feature similarity between the two images is judged to complete image classification. The multi-iris feature recognition matching process is shown in Figure 7.

For the extracted iris fusion feature vectors, the similarity between the iris templates to be matched and the templates in the iris image database is calculated based on the Euclidean distance, and a preset threshold is compared to determine whether the two iris images come from the same eye. The calculation of Euclidean distance is given by the formula:


$$\begin{array}{l} \text{Eudist}=\sqrt{\overset{n}{\underset{i=1}{\sum }}{\left(a_{i}-b_{i}\right)}^{2}} \end{array}$$ (9)

where *a*_*i*_ and *b*_*i*_ represent points in feature spaces *X* and *Y*, respectively. If the distance exceeds the threshold, the iris to be recognized does not match the target class; on the contrary, if the distance is less than or equal to the threshold, it is considered to match the target class.

### 4.4 Design of feature fusion recognition algorithm

**Figure 8:**
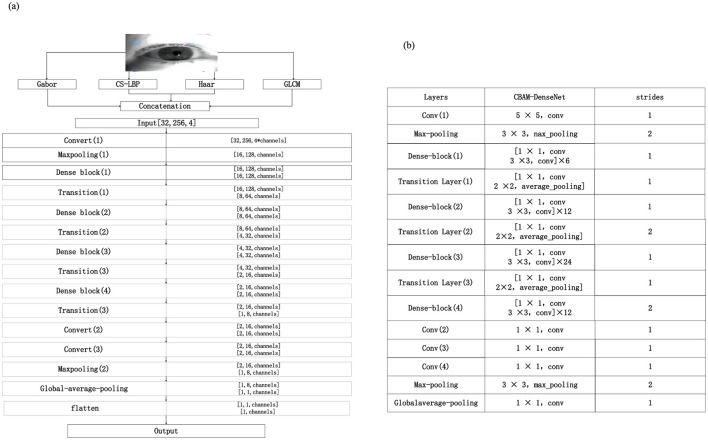
CBAM-DenseNet network model and structural parameters. **(a)** CBAM-DenseNet network model. **(b)** Parameters of the CBAM-DenseNet network structure.

For the problem of iris recognition with multi-feature fusion, a CBAM-DenseNet network structure is proposed, which integrates frequency domain, wavelet decomposition mapping, global texture analysis, and spatial domain features of iris images to enrich the image information input to the network model. The existing DenseNet network structure has been optimized to be more suitable for multi-feature feature extraction and recognition of irises. Figure 8a shows the proposed CBAM-DenseNet network model and its detailed parameter settings. The relevant hyperparameters of the CBAM-DenseNet network are set using the Adam optimizer as shown in Table 2. The network model structure settings adopted are shown in Figure 8b.

**Table 2:** Hyperparameter settings.

**Hyperparameter**	**Value**
Learning rate	1 × 10^−1^
Growth k	24
Batch size	8
Momentum	0.9
Epochs	50

## 5 Experiment

### 5.1 Data description

**Figure 9:**
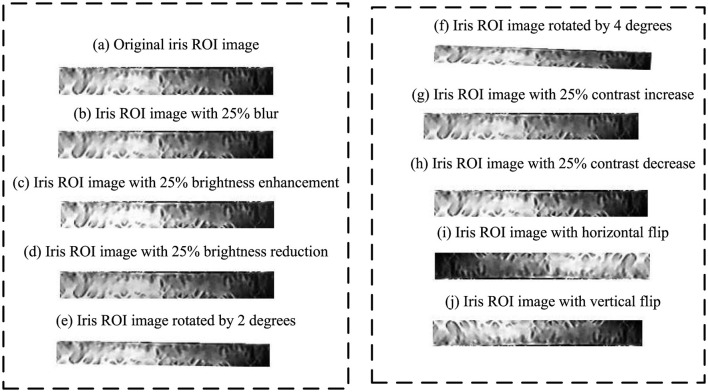
Augmented iris ROI region images.

The experiments utilize the CASIAv4.0, NICE1.0, and JLU-6.0 iris datasets as data sources. Additionally, it incorporates different structural iris types from the CASIAv4.0, including CASIA-IrisV4-Interval (interval) and CASIA-IrisV3-Lamp (visible light). By employing the aforementioned iris image quality assessment methods and preprocessing techniques, iris images from multiple datasets are selected and normalized to construct the iris image dataset, as detailed in Table 3. Additionally, this paper performs data augmentation on iris ROI images, including methods such as image blurring, brightness alteration, image rotation, contrast alteration, and image flipping. The specific operations involve: applying 20% image blurring, brightness variation (25% increase and decrease), image rotation (2° and 4°), contrast variation (25% increase and decrease), and image flipping (horizontal and vertical directions) to augment the iris ROI images. The experimental dataset after augmentation is shown in Figure 9.

**Table 3:** Custom iris dataset details.

**Iris Database**	**Category Count**	**Expanded Class Instances**	**Total**
Casia-IrisV4-interval	100 categories	70 images	7,000 images
Casia-IrisV3-lamp	100 categories	70 images	7,000 images
NICE1.0	48 categories	50 images	2,400 images
JLU-6.0	50 categories	500 images	25,000 images

### 5.2 Analysis of iris image quality evaluation and screening effects

To verify the quality evaluation and screening capabilities of the multi-metric iris image evaluation coefficients and the SA-SVM classifier, the CASIAv4.0 iris image database is utilized. Quality grading is performed through subjective pre-classification by personnel, and the proposed quality evaluation scheme is validated. The main verification includes two aspects: First, the capability of the iris image quality evaluation scheme is analyzed by assessing the accuracy of iris image quality classification; second, the impact of the evaluation scheme on iris recognition accuracy is explored through a pilot experiment with a small sample. The specific steps are as follows:

1. Randomly select about 100 classes of left and right iris images, totaling 2000 images, from the CASIAv4.0 iris image database.
2. Apply the Benner function to 1000 iris images for clarity evaluation, using the median value of the clarity function of these 1000 images as the threshold for classification: images above the threshold are categorized as clear images, and those below are categorized as blurry images, resulting in two main categories including 846 clear images and 154 blurry images. Blurry iris images that do not meet the clarity quality requirements are excluded, yielding 846 clear iris images to construct the experimental dataset IRISQuality.
3. The remaining 846 iris images are categorized according to iris quality evaluation standards into 214 high-quality images, 454 acceptable-quality iris images, and 178 poor-quality iris images.

Based on the experimental dataset IRIS-Quality, iris images of different quality levels are randomly assigned to the training and testing sets at a ratio of 4:1 and input into the SA-SVM classification model for training and learning. Meanwhile, the classification accuracy of this image quality evaluation scheme is compared with other image quality evaluation schemes, with the results shown in Table 4. To evaluate the impact of iris image quality assessment schemes on recognition performance, recognition rate is adopted as the evaluation criterion. Multiple quality assessment methods are used to select iris images for recognition, and it is verified whether the selected images improve matching accuracy. In the preprocessing stage, the Gabor algorithm is used to enhance the ROI area and extract features. The Hamming distance between sample feature images is calculated to determine iris matching. For each method, the Correct Recognition Rate (CRR) and Equal Error Rate (EER) metrics are used for testing, with results detailed in Table 5.

**Table 4:** Comparison of classification performance of image quality evaluation schemes.

**Scheme**	**TAR (%)**	**FAR (%)**	**EER (%)**	**F1-score**	**Accuracy (%)**
Proposed Method	99.2	0.3	0.75	0.991	98.936
SVM	92.1	9.1	5.80	0.912	91.444
GA-SVM	98.9	0.5	0.82	0.988	98.804
BP	93.0	8.8	6.10	0.915	92.180
GA-BP	98.7	0.6	0.88	0.986	98.620

**Table 5:** Comparison of computational efficiency and resource consumption of different quality assessment schemes.

**Scheme**	**Inference (ms)**	**Model (MB)**	**GPU (MB)**	**Training (min)**
Proposed Method	4.2	12.8	320	28
SVM	1.8	3.5	150	15
GA-SVM	2.1	4.1	160	42
BP	3.5	8.7	280	35
GA-BP	3.9	9.2	290	68

Analysis results indicate that the multi-metric iris image quality assessment scheme based on the SA-SVM model can effectively evaluate iris quality and filter out low-quality irises, thereby enhancing recognition accuracy. Moreover, after screening, the match between iris images and feature extraction and recognition algorithms is improved, significantly increasing the success rate of iris recognition, thus having a positive effect on iris identification.

### 5.3 Evaluation of multi-feature fusion effects

**Figure 10:**
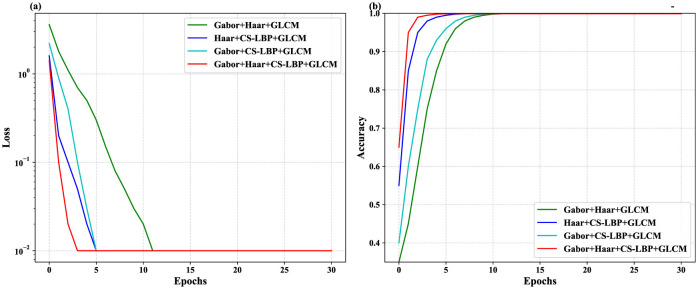
Comparison of loss and accuracy after training with different feature fusion schemes. **(a)** Comparison of loss for different feature fusion schemes post-training. **(b)** Comparison of accuracy for different feature fusion schemes post - training.

In the preliminary experiment, 100 classes of iris images from different eyes in the CASIA-v4.0 iris database were selected. After image quality assessment and screening, 20 iris images per class were obtained, totaling 2,000 iris images. These images were preprocessed to yield 1,000 iris ROI images of size 32 × 256 as the source of experimental data. The experimental data were divided into training and testing sets with a ratio of 8/2. Different iris feature fusion schemes were set up and the iris features were re-extracted and fused based on the optimized CBAM-DenseNet network. Further analysis was conducted on the impact of different iris feature fusions on the recognition accuracy of the network and the loss of data during the training process to select the appropriate iris feature fusion scheme. The experimental data were subjected to feature extraction and fusion according to Schemes 1 to 4. The resulting fused iris feature images were input into the CBAM-DenseNet network for training, and the results obtained from the training data are summarized in Figure 10.

Summary of the experimental results indicates that the iris feature fusion images from Scheme 4 exhibit faster convergence, higher recognition accuracy, and better stability within neural networks. This is attributed to the integration of various iris feature extraction methods in Scheme 4, which enhances the accuracy and stability of iris recognition, thus being selected as the optimal fusion scheme.

### 5.4 Evaluation of multi-feature fusion recognition effects

In addressing the problem of image classification and recognition, this experiment compares the performance of various mainstream iris image recognition and classification models currently available. The focus of this experiment is to explore the recognition accuracy (CRR) of different iris recognition and classification models on different types of iris databases. By comparing the experimental results with other models, the advantages of the proposed iris recognition model in the field of iris recognition are discussed. The proposed iris multi-feature fusion recognition model was validated on the four datasets mentioned in Section 5.1: CASIAIrisV4-Interval, CASIA-IrisV3-Lamp, NICE1.0, and JLU-6.0, with the results presented in Table 6. Comparative analysis indicates that deep learning-based iris recognition algorithms outperform in accuracy. Despite the CASIA and NICE databases being larger, their performance was lower than expected when compared to the JLU6.0 dataset with a larger sample size. The proposed multi-feature fusion iris model in this study achieved superior recognition accuracy across four datasets, thereby validating its effectiveness. Image quality and size from different databases affect accuracy rates. DenseNet and its variants excel in small-sample sets, underscoring the importance of dataset selection and quality. Moreover, the proposed model leverages the feature extraction advantages of DenseNet and integrates multiple stable iris features, enriching input data and enhancing classification performance.

**Table 6:** Average recognition accuracy (CRR) based on different experimental datasets.

**Recognition model**	**CASIA-IrisV4-Interval**	**CASIA-IrisV3-Lamp**	**NICE1.0**	**JLU-6.0**
AlexNet	71.46%	68.67%	80.12%	83.28%
ResNet101	84.00%	83.33%	83.00%	87.13%
DenseNet121	88.42%	86.43%	74.56%	89.45%
SE-DenseNet	90.16%	88.77%	85.00%	92.15%
CBAM-DenseNet	93.46%	90.15%	89.74%	96.70%

### 5.5 Ablation study

To validate the contribution of each component in our proposed framework, we conduct a systematic ablation study on the NICE1.0 dataset–the same benchmark used in Section 5.4. We evaluate four progressively enhanced configurations: (1) a baseline DenseNet-121 model; (2) DenseNet-121 augmented with Dropout and standard data augmentation for regularization; (3) the model further integrated with the CBAM attention module at the final dense block; and (4) the full proposed model incorporating multi-feature fusion (combining Gabor, LBP, Haar, and CNN features). All variants are trained and evaluated under identical settings to ensure a fair comparison. The results are summarized in Table 7.

**Table 7:** Ablation study on the NICE1.0 dataset.

**Model configuration**	**Key components**	**Accuracy**
(1) Baseline model	DenseNet-121	74.63%
(2) + Regularization	+ Dropout & data augmentation	79.85%
(3) + Attention	+ CBAM module	85.37%
(4) Full model	+ Multi-feature fusion	89.74%

As shown, the baseline DenseNet-121 achieves an accuracy of 74.63%, reflecting the challenging nature of the NICE1.0 dataset due to significant noise and occlusion. Introducing regularization improves performance by 5.2 percentage points (to 79.85%), demonstrating its effectiveness in mitigating overfitting under limited training data. The addition of the CBAM attention mechanism yields a substantial gain of 5.50%, raising accuracy to 85.37%, which highlights its ability to suppress irrelevant background regions and enhance discriminative iris textures. Finally, integrating handcrafted features via multi-feature fusion further boosts performance to 89.74%, aligning closely with the 89.74% reported for CBAM-DenseNet in Table 4. This consistency confirms the reliability of our experimental analysis and underscores the complementary benefits of combining deep and traditional features.

## 6 Conclusion

This paper conducts an in-depth study on iris recognition technology, aiming to design a multi-feature fusion-based iris recognition algorithm, and validates its effectiveness and performance. The focus is on the quality assessment and preprocessing of iris images, proposing a feature re-extraction method based on CBAM-Densenet. Experimental results demonstrate that the multi-feature fusion scheme effectively enhances iris image quality, thereby significantly improving the accuracy and robustness of iris recognition.

## Data Availability

The original contributions presented in the study are included in the article/supplementary material, further inquiries can be directed to the corresponding author.
